# Virulence Factors and O-Serogroups Profiles of Uropathogenic *Escherichia Coli* Isolated from Iranian Pediatric Patients

**DOI:** 10.5812/ircmj.14627

**Published:** 2014-02-03

**Authors:** Banafshe Dormanesh, Farhad Safarpoor Dehkordi, Sahar Hosseini, Hassan Momtaz, Reza Mirnejad, Mohammad Javad Hoseini, Emad Yahaghi, Vahideh Tarhriz, Ebrahim Khodaverdi Darian

**Affiliations:** 1 Department of Pediatric Nephrology, AJA University of Medical Sciences, Tehran, IR Iran; 2 Young Researchers and Elites Club, Islamic Azad University, Shahrekord Branch, Shahrekord, IR Iran; 3 Department of Microbiology, College of Veterinary Medicine, Islamic Azad University, Shahrekord Branch, Shahrekord, IR Iran; 4 Molecular Biology Research Center, Baqiyatallah University of Medical Sciences, Tehran, IR Iran; 5 Young Researchers and Elite Club, North Tehran Branch, Islamic Azad University, Tehran, IR Iran; 6 Department of Pharmaceutical Biotechnology, Faculty of Pharmacy, Tabriz University of Medical Sciences, Tabriz, IR Iran; 7 Young Researchers and Elite Club, Islamic Azad University, Karaj Branch, Karaj, IR Iran

**Keywords:** Uropathogenic *Escherichia Coli*, Pediatrics, Iran

## Abstract

**Background:**

Uropathogenic *Escherichia coli* O- Serogroups with their virulence factors are the most prevalent causes of UTIs.

**Objectives:**

The present investigation was performed to study the virulence factors and O-Serogroups profiles of UPEC isolated from Iranian pediatric patients.

**Patients and Methods:**

This cross sectional investigation was performed on 100 urine samples collected from hospitalized pediatrics of Baqiyatallah Hospital, Tehran, Iran. Midstream urine was collected to decrease potential bacterial, cellular and artifactual contamination. All samples were cultured and those with positive results were subjected to polymerase chain reactions to detect pap, cnf1, afa, sfa and hlyA genes and various O- Serogroups.

**Results:**

We found that 37.5% of boys and 75% of girls had positive results for Escherichia coli. We also found that O1 (19.33%), O2 (13.33%), O6 (13.33%), O4 (11.66%), and O18 (11.66 %) were the most commonly detected Serogroups. Totally, the serogroup of 5% of all strains were not detected. In addition, all of these O- Serogroups were pap+, cnf1+, hlyA+, and afa+. Totally, pap (70 %), cnf1 (56.66 %), and hlyA (43.33 %) were the most commonly detected virulence genes in the both studied groups of children. The sfa (30 %) and afa (26.66 %) genes had the lowest incidence rates.

**Conclusions:**

Special health care should be performed on UTIs management in Iranian pediatric patients. Extended researches should be performed to evaluate relation between other O-Serogroups and virulent genes.

## 1. Background

Despite all advances in medical sciences, UTIs remains as one of the most prevalent infectious diseases worldwide leading to severe disorders like cystitis and pyelonephritis (1). The average incidence rate of febrile UTIs is about 1% in boys and 3-8% in girls (2, 3). The Uropathogenic Escherichia coli (E. coli (UPEC)) strains are the most common causes of UTIs (4). Evaluating the potential virulence genes is required to assess the pathogenicity of UPEC strains in UTIs. Successful colonization, establishment, and ultimately leading to UTIs by UPEC strains is based on the ability to adhere to host surfaces such as mucous membranes, urinary epithelial or kidney tissue. The most important virulence factors in UPEC strains are P fimbriae (pap), a fimbrial adhesin I (afaI), hemolysin (hly), cytotoxic necrotizing factor 1 (cnf 1), and S fimbriae (sfa) (1, 4-6) . Among all 174 detected *E. coli* O-Serogroups O7, O2, O4, O15, O6, O21, O1, O25, O16, O22, O8, O75, O18, and O83 Serogroups were previously isolated from the severe cases of UTIs (1, 4, 7-10). Unfortunately, there is a limited published data about the distribution of virulence genes and O-Serogroups in UPEC strains isolated from pediatrics with UTIs in Iran.

## 2. Objectives

This study was performed to study the distribution of virulence genes and serogroups among Iranian pediatrics with UTIs.

## 3. Materials and Methods

### 3.1. Samples Collection and Escherichia coli Identification

This cross sectional study was performed from October to December 2012. A total of 100 urine samples were collected from patients with UTIs. All of patients were younger than 3 years. All samples were collected from the hospitalized pediatrics of Baqiyatallah Governmental Hospital in Tehran, Iran. Midstream urine was collected in sterile condition to decrease potential bacterial, cellular and artifactual contamination. All samples were immediately transferred to the Biotechnology and Microbiology Research Center of the Islamic Azad University at 4°C. Totally, 3 mL of each sample was blended with 225 mL of nutrient broth (Merck, Germany) for 2 min at normal speed, using a Stomacher lab blender and incubated at 37 °C for 24h. One milliliter sample of the nutrient broth culture was mixed with 9 mL of MacConkey broth (Merck, Germany) and further incubated at 37 °C for 24h. One loop of each tube was streaked on MacConkey agar (Merck, Germany). A typical purple colony of E. coli was streaked on Eosin Methylene Blue agar (EMB agar) plate (Merck, Germany) and incubated at 37 °C for 24h. A metallic green colony from each plate with typical E. coli morphology was selected and examined by biochemical tests, including hydrogen sulfide, citrate, urease and indole.

### 3.2. DNA Isolation and E. coli Confirmation

Bacterial strains were sub cultured overnight in Luria-Bertani broth (Merck, Germany) and genomic DNA was extracted from typical colonies of E. coli using DNA extraction kit (DNPTM, CinnaGen, Iran) according to manufacturer’s instruction. All of the positive colonies were confirmed using the polymerase chain reaction (PCR) technique. Table 1 shows the list of primers used for detection of 16SrRNA gene of *E. coli* strains isolated from pediatrics. PCR was performed with a total volume of 50 µL including 2 mM MgCl2, 1 µM of forward primer, 1 µM of reverse primer, 5 µL PCR buffer 10X, 200 µM dNTP (Fermentas), 1 U Taq DNA polymerase (Fermentas) and 2.5 µL DNA template. The DNA was then amplified by 31 successive cycles of denaturation at 95°C for 45s, primer annealing at 59°C for 60s, and DNA chain extension at 72°C for 60s.

**Table 1.** Used Primers for O-Serogroups Amplification in Uropathogenic *Escherichia Coli* Strains Isolated from Pediatric Patients (Li et al. 2010) (11)

Serogroup	Target Gene	Primer Name	Primer Sequence (5’-3’)	Size of Product, bp
**O1**	wzx	Wl-14632	GTGAGCAAAAGTGAAATAAGGAACG	1098
		wl-14633	CGCTGATACGAATACCATCCTAC	
**O6**	wzy	wl-14646	GGATGACGATGTGATTTTGGCTAAC	783
		wl-14647	TCTGGGTTTGCTGTGTATGAGGC	
**O7**	wzx	wl-14648	CTATCAAAATACCTCTGCTGGAATC	610
		wl-14649	TGGCTTCGAGATTAAACCTATTCCT	
**O8**	orf469	wl-14652	CCAGAGGCATAATCAGAAATAACAG	448
		wl-14653	GCAGAGTTAGTCAACAAAAGGTCAG	
**O16**	wzx	wl-14654	GGTTTCAATCTCACAGCAACTCAG	302
		wl-14655	GTTAGAGGGATAATAGCCAAGCGG	
**O21**	wzx	wl-14676	CTGCTGATGTCGCTATTATTGCTG	209
		wl-14677	TGAAAAAAAGGGAAACAGAAGAGCC	
**O75**	wzy	wl-17413	GAGATATACATGGGGAGGTAGGCT	511
		wl-17414	ACCCGATAATCATATTCTTCCCAAC	
**O2**	wzy	wl-14636	AGTGAGTTACTTTTTAGCGATGGAC	770
		wl-14637	AGTTTAGTATGCCCCTGACTTTGAA	
**O4**	wzx	wl-14642	TTGTTGCGATAATGTGCATGTTCC	664
		wl-14643	AATAATTTGCTATACCCACACCCTC	
**O15**	wzy	wl-14672	TCTTGTTAGAGTCATTGGTGTATCG	183
		wl-14673	ATAAAACGAGCAAGCACCACACC	
**O18**	wzx	wl-14656	GTTCGGTGGTTGGATTACAGTTAG	551
		wl-14657	CTACTATCATCCTCACTGACCACG	
**O22**	wzx	wl-14660	TTCATTGTCGCCACTACTTTCCG	468
		wl-14661	GAAACAGCCCATGACATTACTACG	
**O25**	wzy	wl-14666	AGAGATCCGTCTTTTATTTGTTCGC	230
		wl-14667	GTTCTGGATACCTAACGCAATACCC	
**O83**	wzx	wl-14668	GTACACCAGGCAAACCTCGAAAG	362
		wl-14669	TTCTGTAAGCTAATGAATAGGCACC	
***E. coli***	16SrRNA	wl-3110	AGAGTTTGATCMTGGCTCAG	919
		wl-3111	CCGTCAATTCATTTGAGTTT	

### 3.3. O-Serogroups Amplification

Presences of various uropathogenic O-Serogroups (O1, O4, O2, O7, O6, O15, O8, O21, O25, O16, O22, O75, O18, and O83) in UPEC strains were investigated using the PCR techniques. Used primers for O- Serogroups amplification is shown in Table 1. The PCR methods for amplification of O1, O7, O6, O21, O16, and O75 Serogroups was performed with a total volume of 50 µL including 2.5 mM MgCl2, 0.4 µM of forward primer, 0.4 µM of reverse primer, 0.4 µL PCR buffer 10X, 300 µM dNTP (Fermentas), 2 U Taq DNA polymerase (Fermentas), and 3 µL DNA template. The DNA was then amplified by 30 successive cycles of denaturation at 95°C for 30s, primer annealing at 55°C for 60s, and DNA chain extension at 72°C for 60s. Also, The PCR methods for amplification of O2, O4, O15, O18, O22, O25, and O83 serogroups was performed with a total volume of 50 µL including 2.5 mM MgCl2, 0.6 µM of forward primer, 0.6 µM of reverse primer, 0.4 µL PCR buffer 10X, 300 µM dNTP (Fermentas), 1.5 U Taq DNA polymerase (Fermentas), 3 µL DNA template, and 3 µL DMSO. The DNA was then amplified by 30 successive cycles of denaturation at 94°C for 60s, primer annealing at 56°C for 60s, and DNA chain extension at 72°C for 90s. The programmable thermal cycler (Eppendorf, Mastercycler® 5330, Eppendorf-Netheler-Hinz GmbH, Hamburg, Germany) PCR device was used in all the PCR reactions.

### 3.4. Virulence Genes Amplification

In the present study the most important virulence genes of UPEC strains including pap, afa, hlyA, cnf 1, and sfa were detected in positive samples. Table 2 shows the list of used primers for detection of UPEC virulence genes. A PCR method was performed with a total volume of 50 µL including 1.5 mM MgCl2, 0.4 µM of forward primer, 0.4 µM of reverse primer, 5 µL PCR buffer 10X, 200 µM dNTP (Fermentas), 1 U Taq DNA polymerase (Fermentas), and 4 µL DNA template. The DNA was then amplified by 30 successive cycles of denaturation at 94°C for 60s, primer annealing at 63°C for 30s, and DNA chain extension at 72°C for 90s with a programmable thermal cycler (Eppendorf, Mastercycler® 5330, Eppendorf-Netheler-Hinz GmbH, Hamburg, Germany). All PCR products were analyzed by electrophoresis (120 V/208 mA) in 1.5% agarose gel and stained by ethidium bromide. A molecular weight marker with 100 bp increments (100bp ladder, Fermentas, Germany) and 1 kbp increments (1000bp ladder, Fermentas, Germany) was used as size standard.

**Table 2.** Used Primers for Virulence Genes Amplification in Uropathogenic Escherichia Coli Strains Isolated from Pediatric Patients (Yamamoto Et Al. 1995) (12) , (Le Bouguenec et al. 1992) (13)

Gene	Primer Name	Primer Sequence (5'-3')	Size of Product, bp
**pap**	pap3	GCAACAGCAACGCTGGTTGCATCAT	336
	pap4	AGAGAGAGCCACTCTTATACGGACA	
**cnf1**	cnf1	AAGATGGAGTTTCCTATGCAGGAG	498
	cnf2	TGGAGTTTCCTATGCAGGAG	
**hlyA**	hly1	AACAAGGATAAGCACTGTTCTGGCT	1177
	hly2	ACCATATAAGCGGTCATTCCCGTCA	
**sfa**	sfa1	CTCCGGAGAACTGGGTGCATCTTAC	410
	sfa2	CGGAGGAGTAATTACAAACCTGGCA	
**afa**	afa1	GCTGGGCAGCAAACTGATAACTCTC	750
	afa2	CATCAAGCTGTTTGTTCGTCCGCCG	

### 3.5. Statistical Analysis

Chi-square test was used to assess any significant correlation between incidences of virulence factors and O-Serogroups of UPEC strains isolated from pediatrics with UTIs using SPSS software (Version 17.SPSS Inc. United States). P value below 0.05 was considered as statistically significant.

### 3.6. Ethical Issues

In the current study, we tried to protect the life, health, dignity, integrity, right to self-determination, privacy, and confidentiality of personal information of research subjects. We conform to generally accepted scientific principles, be based on a thorough knowledge of the scientific literature, other relevant sources of information, and adequate laboratory experimentation. All samples were taken from the patients who were volunteered for this research. All ethical issues were considered, and this research was performed with the hospital permission. In addition, the present study was approved by the ethical committee of Baqiyatallah Hospital, Tehran, Iran and Microbiology and Infectious Diseases Research committee of the Islamic Azad University of Shahrekord Branch, Iran. Written informed consent was obtained from all of the study patients or their parents. The ethical approval was performed on 10 October 2012 (MB612539122).

## 4. Results

**Figure 1.**
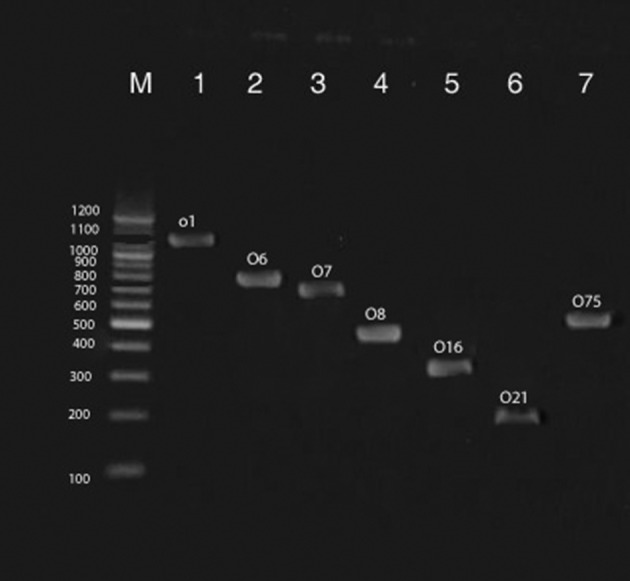
Multiplex PCR Assay for Detection of O1, O6, O7, O8, O16, O21 and O75 Serogroups of Uropathogenic Escherichia Coli Strains Isolated from Pediatrics With UTIs. Line M is 100 bp Ladders; 1-7 Are Positive Samples

**Figure 2.**
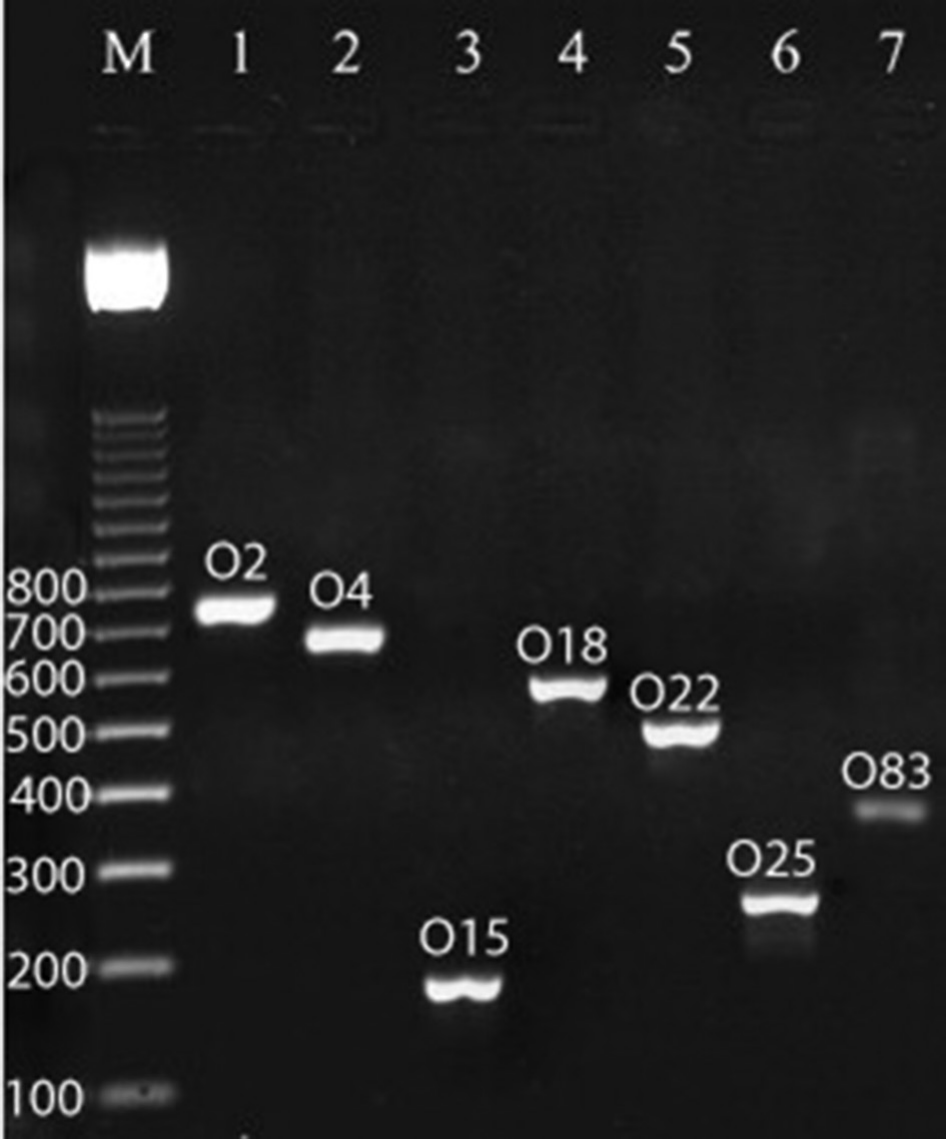
Multiplex PCR Assay for Detection of O2, O4, O15, O18, O22, O25, and O83 Serogroups of Uropathogenic Escherichia Coli Strains Isolated From Pediatrics With UTIs. Line M is 100 bp Ladders; 1-7 Are Positive Samples

**Figure 3.**
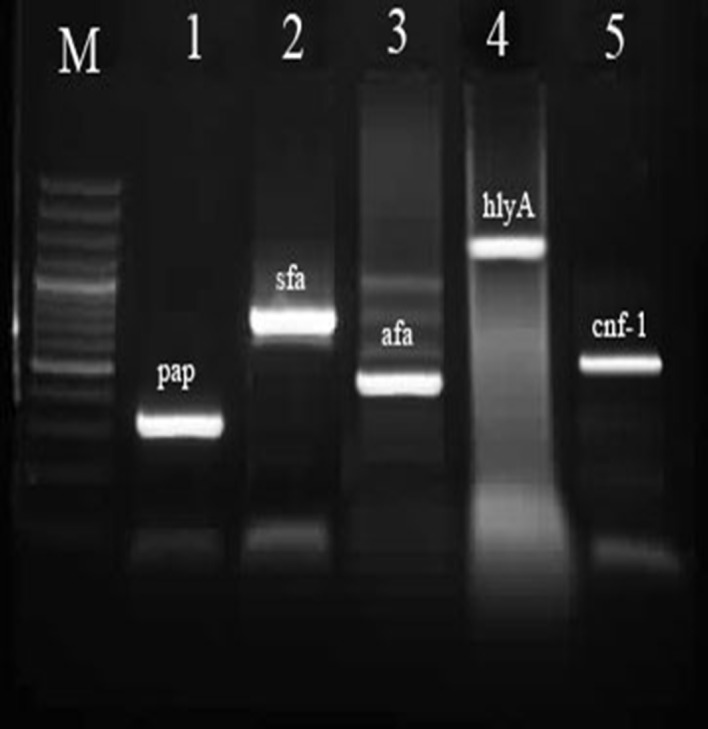
Multiplex PCR Assay for Detection of Virulence Genes of Uropathogenic Escherichia Coli Strains Isolated From Pediatrics With UTIs. Line M is 100 bp Ladder, 1-5 Are Positive Samples.

Sixty of 100 urine samples (60 %) had positive results for E. coli bacterium (Table 3). In addition, 15 of 40 boys urine samples (37.5 %) and 45 of 60 girls urine samples (75 %) had positive results for *E. coli*. All of the samples with positive results were confirmed using the PCR technique. There were significant differences (P < 0.05) in the incidence of UPEC strains between the girls and boys groups. The distribution of O-Serogroups in the UPEC strains isolated from boys and girls pediatric patients is shown in Table 4. We found that O1 (18.33 %) had the highest incidence in urine samples of both studied groups of children, followed by O2 (18.33 %), O6 (13.33 %), O4 (13.33 %), and O18 (11.66 %). There were no positive results for O16, and O83 Serogroups in both studied groups of children (Figures 1 and 2). Our results showed significant differences (P < 0.05) between the incidence of O1 and O15, and also between the incidence of O2 and O6 with O7 and O8. We found that O1 Serogroup had the highest incidence of virulence genes in urine samples of boys, followed by non-detected, O2, O18 and O25 (Table 5 and Figure 3). We also found that O1 Serogroup had the highest incidence of virulence genes in urine samples of girls, followed by O2, O6, O18, O4, and O25 (Table 6). Some of the O- Serogroups of our study were pap+, cnf1+, hlyA+, afa+ and sfa+. Totally, pap was the most commonly detected putative virulence gene in the both studied groups (70 %), followed by cnf1 (56.66 %) and hlyA (43.33 %). The Sfa (30 %) and afa (26.66 %) genes had lower distribution among various O- Serogroups. Statistical analyses showed a significant (P < 0.05) association between the incidence of the pap gene and afa gene in the both studied groups. We also found statistically significant (P <0.05) association in the incidence of all studied virulence genes between O1 and O8 and O15 Serogroups.

**Table 3.** Distribution of Uropathogenic Escherichia Coli Isolated From Pediatric Patients in Iran

Pediatric Patients	No. Samples	Culture Positive, No. ( %)	PCR Confirmation, No. ( %)
**Boy**	40	15 (37.5)	15 (37.5)
**Girl**	60	45 (75)	45 (75)
**Total**	100	60 (60)	60 (60)

**Table 4.** Distribution of Uropathogenic Escherichia Coli Serogroups Isolated From Pediatric Patients in Iran

Pediatric Patients (Positive Samples)	O-Serogroups, No. ( %)														
	O1	O2	O4	O6	O7	O8	O15	O16	O18	O21	O22	O25	O75	O83	Non Detected
**Boy (15)**	3	2 (13.33)	2 (13.33)	2	-	-	-	-	2 (13.33)	-	-	2 (13.33)	1 (6.66)	-	1 (6.66)
**Girl (45)**	(20)	6 (13.33)	5 (11.11)	(13.33)	1 (2.22)	1 (2.22)	2 (4.44)	-	5 (11.11)	3 (6.66)	3 (6.66)	2 (4.44)	1 (2.22)	-	2 (4.44)
**Total (60)**	8 (17.77)	8	7 (11.66)	6 (13.33)	1 (1.66)	1 (1.66)	2	-	7 (11.66)	3 (5)	3 (5)	4	2 (3.33)	-	3 (5)

**Table 5.** Distribution of Putative Virulence Genes in Uropathogenic *Escherichia Coli* O-Serogroups Isolated from Boys Patients in Iran

Gene	O1 (3)	O2 (2)	O4 (2)	O6 (2)	O7 (-)	O8 (-)	O15 (-)	O16 (-)	O18 (2)	O21 (-)	O22 (-)	O25 (2)	O75 (1)	O83 (-)	Non Detected (1)
**Pap (12)**	3	2	1	1	-	-	-	-	2	-	-	2	1	-	-
**cnf1 (9)**	2	1	1	1	-	-	-	-	1	-	-	1	-	-	2
**hlyA (8)**	2	1	1	1	-	-	-	-	1	-	-	1	-	-	2
**Sfa (6)**	2	1	-	-	-	-	-	-	1	1	-	1	-	-	1
**Afa (7)**	2	1	1	1	-	-	-	-	1	-	-	1	-	-	-

**Table 6.** Distribution of Putative Virulence Genes in Uropathogenic Escherichia Coli O-Serogroups Isolated from Girls Patients in Iran

Gene	O1 (8)	O2 (6)	O4 (5)	O6 (6)	O7 (1)	O8 (1)	O15 (2)	O16 (-)	O18 (5)	O21 (3)	O22 (3)	O25 (2)	O75 (1)	O83 (-)	Non Detected (2)
**Pap (30)**	8	5	3	4	1	1	-	-	3	1	1	1	1	-	1
**cnf1 (25)**	6	4	2	3	1	-	1	-	2	1	1	1	1	-	2
**hlyA (18)**	5	3	1	2	-	1	1	-	1	1	1	1	-	-	1
**Sfa (12)**	3	2	-	2	-	-	-	-	1	1	1	1	-	-	1
**Afa (9)**	3	2	1	1	-	-	-	-	-	-	-	1	-	-	1

## 5. Discussion

The results of our investigation showed that girls were more prone to get UTIs than boys. Similar results were reported previously (1, 14-16). It is because of the relatively short, straight anatomy of the urethra in women. In addition, retrograde ascent of bacteria from the perineum is the most common cause of UTIs in women. Genetic factors, including expression of Lewis blood group Le (a+b-) and Le (a-b-) and HLA-A3 may also expose women at a higher risk for recurrent UTIs. Several O-Serogroups have been detected in urine samples of our study. Totally, O1, O2, O4, O6, O18 and O25 were the most commonly detected O- Serogroups in UPEC strains of our study. Similar results have been reported previously (1, 4, 17). Fifty percent of UPEC strains of Molina-Lopez et al. (2011) (18) investigations belonged to Serogroups O2, O1, O6, O4, O25, O75, and O8. Emamghoraishi et al. (2011) (19) reported that from a total of 96 strains of E. coli isolated from urine samples of Iranian children with UTIs, 12.2% were O1, 10.2% were O6 and 4.1% were O15 Serogroups. According to the results of Momtaz et al. (2013) (4), O25 (26.01%), O15 (21.13%), O4 (5.69%), O1 (2.43%), and O2 (2.43%) were the most commonly detected Serogroups among Iranian hospitalized patients. Fathollahi et al. (2009) (16) reported that 66.14% of UPEC strains isolated from Iranian patients with UTIs belonged to O1, O18, O6, O20, and O15 Serogroups. The UPEC strains from a relatively small number of O Serogroups, mainly O1, O2, O4, O6, O18 and O25 were reported to account for a major part of O- Serogroups UTI strains from different parts of the world (1, 4, 16). These Serogroups were isolated from the cases of pyelonephritis and cystitis previously (1, 20).

It has been repeatedly reported that O1, O2, O4, O6, O25, and O18 Serogroups possess specific virulence factors, which confer on their special adhesive and invasive abilities (1, 4). Most O- Serogroups of our study were pap+, cnf1+, hlyA+, sfa+, and afa+. Totally, Pap, cnf1 and hlyA were the most commonly detected virulence genes in our study. Similar Iranian studies have been reported previously by Arabi et al. (2012) (21), Momtaz et al. (2012) (22), Asadi et al. (2012) (23) and Emamghorashi et al. (2011) (19). Ninety two percent of UPEC strains of Arabi et al. (2012) (21) were harbored the fim and sfa genes, separately. Momtaz et al. (2012) (22) reported that the distribution of pap, hlyA, cnf1and afa virulence genes were 50.4%, 50.4%, 50.4%, and 8.13%, respectively. The pap gene was detected in 80% of boys and 66.66% of girls of our study. This gene plays important roles in the pathogenesis of pyelonephritis and ascending UTIs 21. Production of cytokines and adhesion to tissue matrix and mucosa are performed by this gene (24). Attachment of pap gene to the epithelial receptor leads to the release of ceramide, which acts as an agonist of Toll-like receptor 4 (TLR4), a receptor involved in activation of the immune cell response (25). This event leads to the development of pain and local inflammation (26).

The sfa gene was detected in 13.33% of boys and 26.66% of girls in our study. This gene is responsible for adhesion to the endothelial and epithelial cells of the lower urinary tract and kidney tissues (27). The sfa gene has also been detected in the E. coli strains, which causes sepsis, meningitis, and ascending UTIs. The cnf1 gene was detected in 60% of boys and 55.55% girls in our investigation. This gene was produced by 1/3 of all pyelonephritis strains, and may also be involved in kidney damages. Several investigations showed that this gene is responsible for polymorph nuclear phagocytosis and epithelial cells apoptosis (Mills et al. 2000). Also, bladder cell exfoliation and enhancement of bacterial access to underlying tissue are caused by the cnf1 gene (28). The most important secreted virulence factor of UPEC strains is a lipoprotein called hlyA, which is associated with upper UTIs such as pyelonephritis (29, 30). Totally, 53.33% of boys and 40% of girls in our study had positive results for the hlyA gene. This toxin is able to lyse nucleated host cells to better cross mucosal barriers, damage effectors' immune cells, induce the apoptosis of T lymphocytes, neutrophils and renal cells, and gain enhanced access to host nutrients and iron stores (29-33). The afa gene was detected in 46.66% of boys and 20% girls in our study. Clinical findings recommend that UPEC strains with afa adhesins have characters potentially favoring the occurrence of pyelonephritis, recurrent and chronic UTIs (13). In conclusion, this study showed that UTIs especially in girls should be considered. Most examined Serogroups of our study had positive results for putative virulence factors. O1, O2, O6, O4 and O18 Serogroups and pap, cnf1 and hlyA virulence factors had highest frequencies for UPEC strains. Our study was the most widely report of direct detection of virulence factors and O- Serogroups of UPEC strains isolated from pediatric patients in Iran. The high prevalence of O1 Serogroups in children with UTIs and the high percentage of virulent genes in O1 Serogroups suggested a close relation between Serogroups and genotypes of UPEC strains.
